# Activation of the AMPK-SIRT1 pathway contributes to protective effects of Salvianolic acid A against lipotoxicity in hepatocytes and NAFLD in mice

**DOI:** 10.3389/fphar.2020.560905

**Published:** 2020-11-30

**Authors:** Songtao Li, Qianyu Qian, Na Ying, Jianfei Lai, Luyan Feng, Sitong Zheng, Fusheng Jiang, Qing Song, Hui Chai, Xiaobing Dou

**Affiliations:** ^1^ College of Basic Medicine and Public Health, Zhejiang Chinese Medical University, Hangzhou, China; ^2^ Molecular Medicine Institute, Zhejiang Chinese Medical University, Hangzhou, China; ^3^ College of Life Science, Zhejiang Chinese Medical University, Hangzhou, China

**Keywords:** salvianolic acid A, adenosine monophosphate activated protein kinase, sirtuin 1, lipotoxicity, non-alcoholic fatty liver disease

## Abstract

**Background:** Salvianolic acid A (Sal A), a natural polyphenol compound extracted from *Radix Salvia miltiorrhiza* (known as Danshen in China), possesses a variety of potential pharmacological activities. The aim of this study is to determine mechanisms of hepatoprotective effects of Sal A against lipotoxicity both in cultured hepatocytes and in a mouse model of fatty liver disease.

**Methods:** High-fat and high-carbohydrate diet (HFCD)-fed C57BL/6J mice were employed to establish hepatic lipotoxicity in a mouse model. Two doses of Sal A were administered every other day via intraperitoneal injection (20 and 40 mg/kg BW, respectively). After a 10-week intervention, liver injury was detected by immunohistochemical and biochemical analyses. For *in vitro* studies, we used HepG2, a human hepatoma cell line, and exposed them to palmitic acid to induce lipotoxicity. The protective effects of Sal A on palmitic acid-induced lipotoxicity were examined in Sal A-pretreated HepG2 cells.

**Results:** Sal A treatments attenuated body weight gain, liver injury, and hepatic steatosis in mice exposed to HFCD. Sal A pretreatments ameliorated palmitic acid-induced cell death but did not reverse effects of HFCD- or palmitate-induced activations of JNK, ERK1/2, and PKA. Induction of p38 phosphorylation was significantly reversed by Sal A in HFCD-fed mice but not in palmitate-treated HepG2 cells. However, Sal A rescued hepatic AMP-activated protein kinase (AMPK) suppression and sirtuin 1 (SIRT1) downregulation by both HFCD feeding in mice and exposure to palmitate in HepG2 cells. Sal A dose-dependently up-regulated p-AMPK and SIRT1 protein levels. Importantly, siRNA silencing of either AMPK or SIRT1 gene expression abolished the protective effects of Sal A on lipotoxicity. Moreover, while AMPK silencing blocked Sal A-induced SIRT1, silencing of SIRT1 had no effect on Sal A-triggered AMPK activation, suggesting SIRT1 upregulation by Sal A is mediated by AMPK activation.

**Conclusion:** Our data uncover a novel mechanism for hepatoprotective effects of Sal A against lipotoxicity both in livers from HFCD-fed mice and palmitic acid-treated hepatocytes.

## Introduction

Nonalcoholic fatty liver disease (NAFLD) is the most prevalent liver disease and becoming a new health challenge with the prevalence of 20% in the general population, up to 70% in patients with type 2 diabetes, and 65–85% in patients with obesity (Lobstein et al., 2004; Chalasani et al., 2012). More than one-third of American adults are estimated to have NAFLD, and the prevalence of NAFLD in China has increased to 15–40% (Loomba and Sanyal, 2013; Fan et al., 2017). NAFLD represents a continuum of hepatic injuries, which progress from simple hepatic steatosis to nonalcoholic steatohepatitis, with some patients even ultimately progressing to fibrosis, cirrhosis, and liver failure (Angulo, 2002).

Currently, there is no Food and Drug Administration (FDA)-approved drug for NAFLD. Modifying lifestyle, such as weight loss by a combination of decreased caloric intake and increased physical activity, is an effective treatment of NAFLD and is also the most common recommendation by clinicians. However, lifestyle modifications remain difficult to achieve for most patients. Therefore, identifying highly effectual and safe small molecules based on the therapeutic targets of NAFLD is urgently needed and a high public health priority.

*Radix Salvia miltiorrhiza*, which is also termed as “Danshen” in China, is a traditional herb, which has been widely used for the treatment of cardiovascular diseases (Lin et al., 1996). We previously reported that Danshen extracts conferred hepatoprotective activities that involved multiple mechanisms, such as attenuating lipids accumulation in the liver, stimulating fatty acid catabolism, and alleviating cellular oxidative stress-induced liver injury (Ding et al., 2017; Liu et al., 2017; Ren et al., 2017). In addition, Danshen extracts exerted a strong preventive role against 4-hydroxynonenal-induced hepatotoxicity in alcohol-administered mice (Ding et al., 2017). Salvianolic acid A (Sal A) is a key bioactive component isolated from the root of Danshen (Lian-Niang et al., 1984). Sal A is a phenolic carboxylic acid derivative with a chemical formula name of (2R)-3-(3, 4-Dihydroxyphenyl)-2-[(E)-3-[2-[(E)-2-(3, 4-dihydroxyphenyl) ethenyl]-3, 4-dihydroxyphenyl] prop-2-enoyl] oxypropanoic acid (Oh et al., 2011). Several studies have reported that Sal A possesses a variety of pharmacological properties, including anti-oxidant, anti-inflammatory, anti-fibrotic, and anti-carcinogenic activities (Zhang et al., 2014; Dai et al., 2015; Chien et al., 2016). Sal A has been reported to protect against high-fat diet (HFD)-induced NAFLD by ameliorating hepatic lipid accumulation and inflammation (Ding et al., 2016). Additionally, Sal A was reported to prevent the pathological progression of hepatic fibrosis in HFD-fed and streptozocin-induced diabetic rats (Qiang et al., 2014).

Lipotoxicity, termed as cellular dysfunction induced by an ectopic deposition of lipids in non-adipose tissues, such as hepatocytes, skeletal muscle cells, and pancreatic β-cells, plays an important role in the pathological progression of NAFLD (van Herpen and Schrauwen-Hinderling, 2008). Palmitate acid (16:0), the most abundant saturated fatty acid in foods and inside of the body, is commonly used as an inducer of hepatic lipotoxicity in cultured cells (Ricchi et al., 2009). Endoplasmic reticulum stress and oxidative stress are two well-established mechanisms underlying palmitic acid-triggered lipotoxicity in a variety of cell types, including hepatocytes. However, up-regulation/activation of several other molecular targets may also protect against hepatic lipotoxicity. For instance, adenosine monophosphate activated protein kinase (AMPK) is down-regulated in both HFD-induced liver injury and saturated fatty acids (SFAs)-induced hepatocytes cell death (You et al., 2004; Meng et al., 2015). Moreover, activation of AMPK by either chemical activators or calorie restriction markedly protected against hepatic lipotoxicity in both experimental animal liver and cultured hepatocytes (Chen et al., 2018; He et al., 2020). In addition, up-regulation of SIRT1 played a potential therapeutic role against palmitate-induced hepatic cell death (Li et al., 2017). However, little is known about specific mechanisms mediating the protective effects of Sal A on lipotoxicity-induced liver injury.

In the present study, we investigated the protective mechanisms of Sal A against hepato-lipotoxicity in high-fat and high-carbohydrate diet (HFCD)-fed mice and palmitic acid-treated HepG2 cells. We provided strong evidence that Sal A reversed HFCD-induced liver injury and palmitate-triggered hepatocytes’ cell death. We furthered uncovered that the AMPK-SIRT1 pathway mediates anti-lipotoxic effects of Sal A. The present study contributed additional knowledge about mechanisms contributing to the known beneficial effects of Sal A.

## Materials and Methods

### Animal Model and Experimental Protocol

All experiments described in this study were performed in accordance with the guidelines for animal experiments released by the National Institute of Animal Health. This study was approved by the Animal Ethic Committee of Zhejiang Chinese Medical University. C57BL/6J mice (8-week-old, male) were group-housed in cages in a temperature-controlled vivarium (22 ± 2°C) and maintained on a 12-h light/dark cycle. Animals were divided into four groups (*n* = 12 per group) as follows: normal diet (ND) group, HFCD group, HFCD with 20 mg/kg BW Sal A (HFCD-LS) group, and HFCD with 40 mg/kg BW Sal A (HFCD-HS) group. ND group were maintained on an AIN-93G diet. HFCD group were maintained on a high-fat diet (60% fat, D12492, Research Diets, New Brunswick, NJ) and given water enriched with high-fructose corn syrup equivalent. A total of 42 g/L of carbohydrates was mixed in drinking water at a ratio of 55% fructose (Acros Organics, Morris Plains, NJ) and 45% sucrose (Sigma-Aldrich, St. Louis, MO) by weight. Sal A was dissolved in sterilized physiologic saline with a stock concentration of 20 mg/ml. A total volume of 100 μl Sal A diluted solution or sterilized physiologic saline was given by intraperitoneal injection every other day, respectively. Animals were provided *ad libitum* access to these diets and water for 10 weeks. Food and water intake were recorded daily, and body weight was recorded weekly. At the end of the experiment, mice were anesthetized with sodium pentobarbital (50 mg/kg BW) after an overnight fast. Plasma and liver tissues were harvested for further assays. Alanine aminotransferase (ALT), triglyceride (TG), glycerol, free fatty acids (FFA), total cholesterol, high-density lipoprotein-cholesterol (HDL-C), and low-density lipoprotein-cholesterol (LDL-C) levels were determined by commercial kits from Nanjing Jiancheng Bioengineering Institute (Nanjing, China) according to the manufacturer's instructions. Small pieces of fresh liver were fixed immediately in 10% buffered formalin. After paraffin embedding, 5 μm sections were deparaffinized in xylene and were rehydrated through a series of decreasing concentrations of ethanol. Sections were stained with hematoxylin and eosin (H&E) using a commercial kit (Nanjing Jiancheng Bioengineering Institute, Nanjing, China). Alternatively, portions of fresh liver were flash-frozen and cryostat sections were cut and prepared for staining with Oil Red O. Pathological sections were observed under Nikon eclipse Ti-S fluorescence microscope (Nikon, Tokyo, Japan).

### Chemicals

Sal A was obtained from Chengdu mansite bio-technology solarbio Co., Ltd (Sichuan, China). Palmitic acid-BSA conjugates were prepared as described previously (Li et al., 2014). Briefly, palmitic acid (Sigma Aldrich, St. Louis, MO) was dissolved in ethanol (Sigma Aldrich, St. Louis, MO) and saponified with sodium hydroxide. The sodium salt was dried, re-suspended in saline, and heated at 80°C until completely dissolved. While the solution was still warm, isovolumetric 20% (w/v) BSA (Sigma Aldrich, St. Louis, MO) was added and the mixture was stirred at 50°C for 4 h to allow palmitic acid to bind to BSA. The palmitic acid-BSA complex (3 mmol/L fatty acid: 1.5 mmol/L BSA; molar ratio, 2:1) was then sterilized by filtering and aliquoted for future use. In all the experiments, the control group was exposed to an equal amount of solvent (e.g., BSA, DMSO). DMSO was obtained from Sigma Aldrich (St. Louis, MO).

### Cell Culture

The human hepatoma cell line (HepG2) was obtained from Shanghai Institute of Cell Bank (Shanghai, China). Cells were cultured in Dulbecco’s Modified Eagle Medium (DMEM, Thermo Scientific Inc., VA) containing 10% (v/v) fetal bovine serum (FBS, Biological Industries, Israel), 100 U/ml penicillin and streptomycin (Thermo Scientific Inc, VA) at 37°C in a humidified O_2_/CO_2_ (19:1) atmosphere. Cells were seeded in 24- or 96-well culture plate with 1 ml and 200 μl medium in final, respectively. Sal A was dissolved in DMEM with a stock concentration of 10 mM. A total volume of 10 μl Sal A diluted solution was added to the cells.

### RNA Interference

Cells were transfected with special siRNA for human SIRT1 and AMPK (Santa Cruz Biotechnology, CA) using Lipofectamine 2000 (Thermo Scientific Inc., VA) according to the manufacturer’s instructions. In the control group, cells were transfected with scrambled siRNA (Santa Cruz Biotechnology, CA). Gene silencing efficiency was verified by detecting the protein abundance with immunoblotting.

### Cell Death Assays

Cell death was determined by measurements of lactate dehydrogenase (LDH) release, the 3-(4,5-dimethylthiazol-2-yl)-2,5-diphenyltetrazolium bromide (MTT) test, propidium iodide (PI) staining, and Hoechst staining. For LDH assay, culture medium was collected and analyzed by LDH assay kit (Thermo Scientific Inc., VA) according to the manufacturer’s instructions. For MTT test, cells were incubated with 450 μM MTT (Sigma Aldrich, St. Louis, MO) for 3 h and then centrifuged at 1800 rpm for 10 min at room temperature to remove the supernatant. Afterwards, formazan was extracted from pelleted cells with 600 μl of DMSO for 15 min. The amount of MTT-formazan was determined by 570 nm absorbance with 655 nm as the wavelength reference. For PI staining, cells were trypsinized and stained with PI staining solution (BD Pharmingen, CA) according to the manufacturer’s instructions. Fluorescence was measured by flow cytometry (Accuri c6, BD, CA). For Hoechst staining, cells were stained with Hoechst staining solution (Beyotime Biotechnology, Nantong, China) according to the manufacturer’s instructions after the indicated treatments. The nuclear morphology was imaged by Nikon eclipse Ti-S fluorescence microscope (Nikon, Tokyo, Japan).

### Western-Blot Analysis

Western-blot was performed as described previously (Dou et al., 2018) and the following antibodies were used: phosphorylated-SAPK/JNK (Thr183/Tyr185) rabbit antibody, SAPK/JNK rabbit antibody, phosphorylated-p44/42 MAPK (Erk1/2) (Thr202/Tyr204) rabbit antibody, p44/42 MAPK (Erk1/2) rabbit antibody, phosphorylated-p38 MAPK (Thr180/Tyr182) rabbit antibody, p38 MAPK rabbit antibody, phosphorylated-PKA (Thr197) rabbit antibody, PKA rabbit antibody, phosphorylated-AMPKα (Thr172) rabbit antibody, AMPKα rabbit antibody, SIRT1 rabbit antibody, and GAPDH rabbit antibody (Cell Signaling Technology, Danvers, MA). The antibodies were diluted according to the manufacturer’s instructions at 1: 5,000 for GAPDH and 1:1,000 for the others.

### Statistical Analysis

All experiments were performed in at least three independent experiments and data were expressed as mean ± SD. Student’s *t*-test was used for comparing two unpaired groups, and one-way ANOVA was employed for three or more groups, followed by the post-hoc test with Fisher’s least significant difference (LSD). All statistical analyses were executed using SPSS 17.0. Differences were considered statistically significant when *p* < 0.05.

## Results

Sal A administration ameliorates HFCD-induced body weight gain and increase of plasma lipids.

**FIGURE 1:**
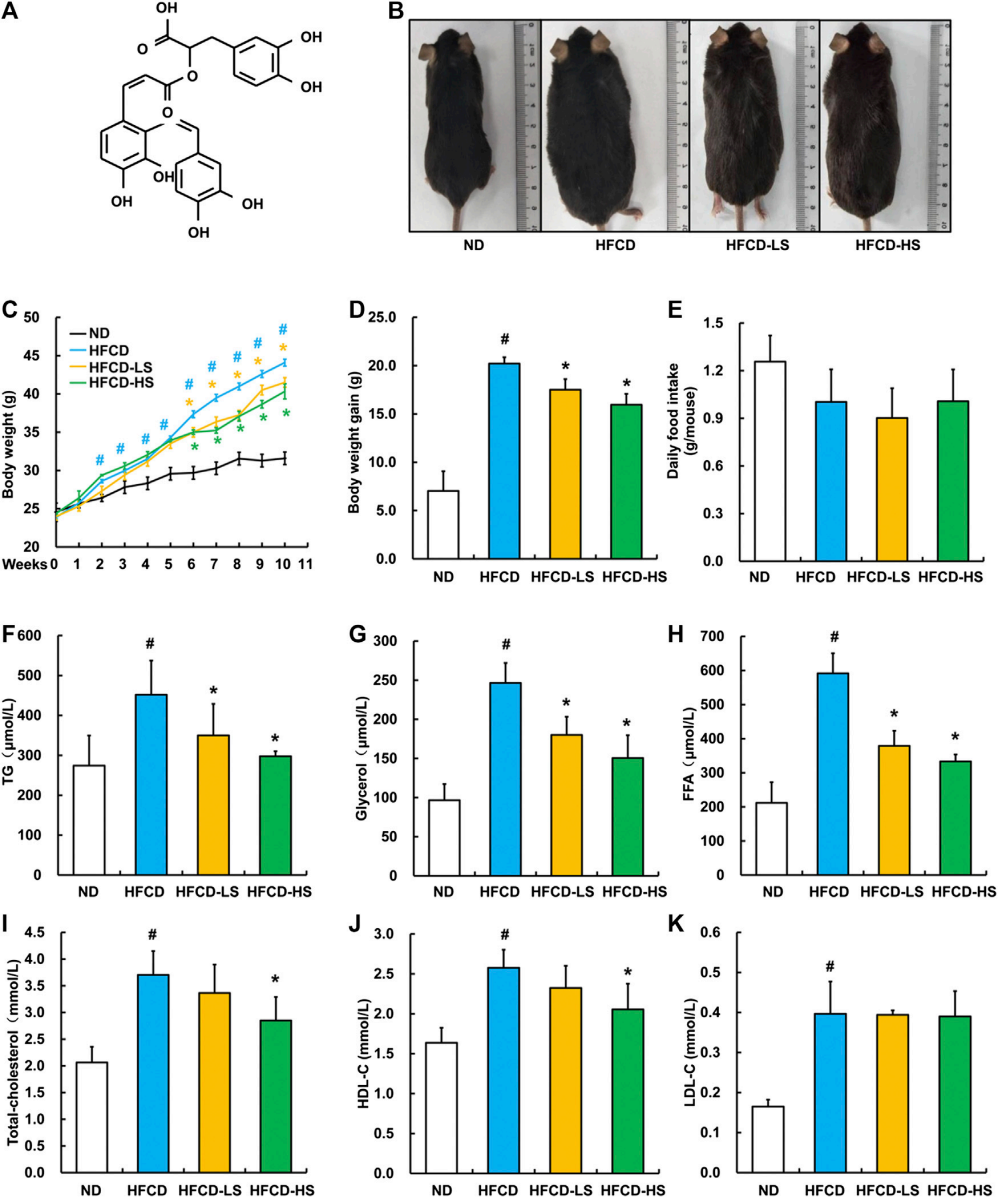
Sal A treatment alleviated HFCD-induced increase in body weight and plasma lipids content in C57bl/6 mice. **(A)** Chemical structures of the Sal A. **(B)** Morphological photographs of the mice. **(C)** Dynamic alteration of body weight. **(D)** Body weight gain. **(E)** Daily food intake. **(F)** Plasma triglycerides (TG) level. **(G)** Plasma glycerol level. **(H)** Plasma free fatty acids (FFA) level. **(I)** Plasma total cholesterol level. **(J)** Plasma high-density lipoprotein-cholesterol (HDL-C) level. **(K)** plasma low-density lipoprotein-cholesterol (LDL-C) level. All values are denoted as means ± SD. The values with different superscripts are significantly different at *p* < 0.05. # Comparisons with normal diet (ND) group; * Comparisons with high-fat diet (HFCD) group. All groups contain 12 animals (*n* = 12).

The chemical structure of Sal A is shown in Figure 1A. After a 13-week HFCD feeding, the body size of mice in HFCD group was obviously larger than that in ND group, whereas Sal A supplementation significantly reversed such alternation (Figure 1B). We did not observe any apparent changes in skin color and hair count among all the groups. In comparison to ND group, mice in HFCD group manifested apparent mobility reduction, which was improved by Sal A administration. Sal A significantly improved HFCD-induced body weight gain (*p* < 0.05, Figures 1C,D) without affecting food intake (*p* > 0.05, Figure 1E). HFCD-induced increases of plasma TG, glycerol, and FFA were significantly rescued by Sal A administration (*p* < 0.05, Figures 1F–H). Although high dose of Sal A reduced HFCD-induced increases of plasma total cholesterol and HDL-C (*p* < 0.05, Figure 1I,J), neither improved HFCD-induced plasma LDL-C increase (*p* > 0.05, Figure 1K).

Sal A administration alleviates hepatic steatosis and liver injury in HFCD-fed mice.

**Figure 2:**
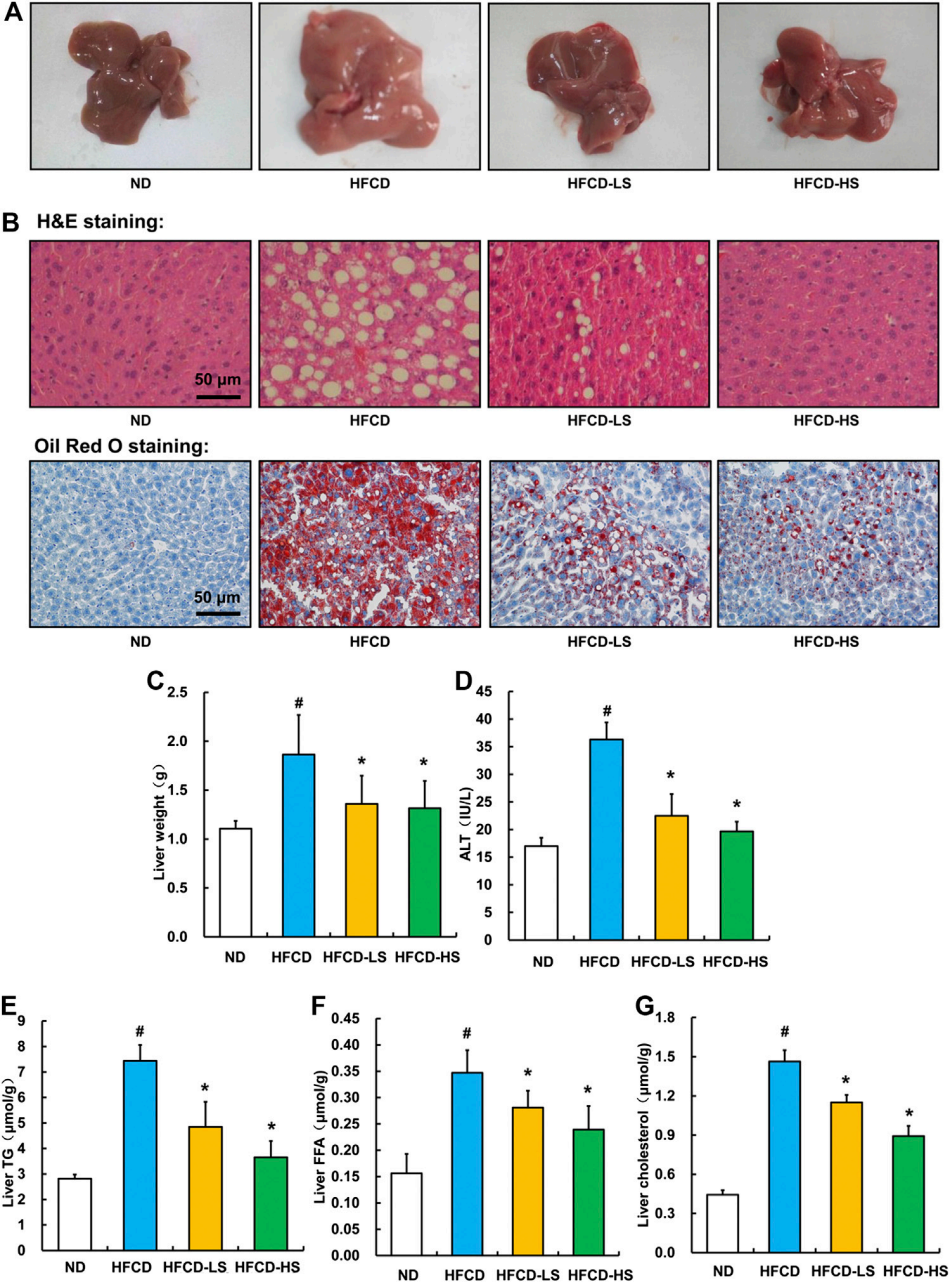
Sal A treatment ameliorated HFCD-induced liver injury and lipids accumulation in live. **(A)** Morphological photographs of livers. **(B)** H&E and oil red O staining photomicrographs of the liver section (200×). **(C)** Liver weight. **(D)** Plasma alanine aminotransferase (ALT) level. **(E)** Triglyceride (TG) content in the liver. **(F)** Free fatty acids level in the liver. **(G)** Total cholesterol level in the liver. All values are denoted as means ± SD. The values with different superscripts are significantly different at *p* < 0.05. # Comparisons with normal diet (ND) group; * Comparisons with high-fat diet (HFCD) group. All groups contain 12 animals (*n* = 12).

Liver pathological alterations were evaluated by morphologic and histological examinations (H&E and oil red O staining). As expected, HFCD-fed markedly increased the liver size and shallowed the liver color (Figure 2A). Massive hepatic steatosis was observed in HFCD group (Figure 2B). Sal A intervention rescued HFCD-induced hepatic pathological alterations mentioned above (Figures 2A,B) and ameliorated HFCD-induced liver weight increment (*p* < 0.05, Figure 2C). The ALT activity, a well-known biomarker of liver injury, was further analyzed. Sal A significantly blunted HFCD-induced plasma ALT elevation (*p* < 0.05, Figure 2D). Moreover, Sal A treatment significantly reduced HFCD-induced increases of TG, FFA, and cholesterol contents in the liver (*p* < 0.05, Figures 2E–G).

Sal A treatment protects against palmitic acid-induced hepatocyte cell death.

**Figure 3:**
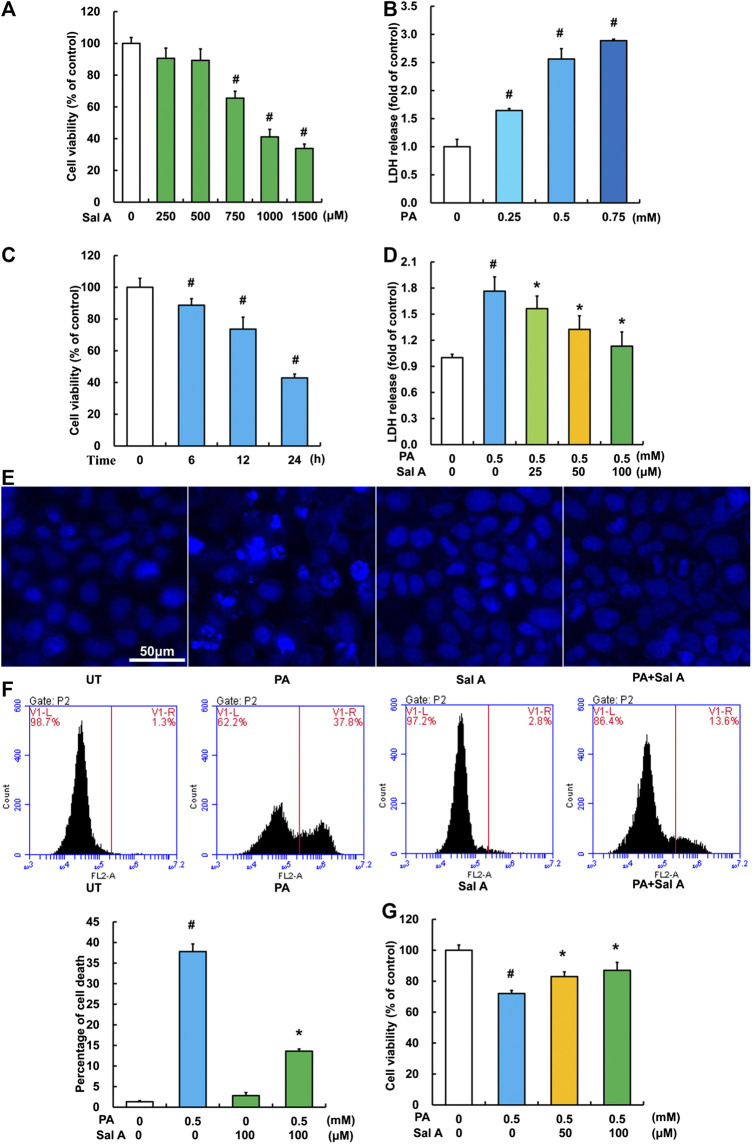
Sal A protected hepatocytes against palmitate-induced cell death. **(A)** HepG2 cells were seeded in 96-well plate and exposed to different doses of Sal A for 24 h. The cytotoxic effect of Sal A was evaluated by MTT test. **(B)** HepG2 cells were cultured in 24-well plate. After 80% confluence, cells were exposed to different doses of palmitic acid (PA) for 12 h. LDH release in the culture medium was detected according to the description in the Material and methods. **(C)** Cells were treated with 0.5 mM PA for 6, 12, and 24 h, respectively. Cell viability was detected by MTT test. **(D)** HepG2 cells were treated with 0.5 mM PA for 12 h with or without 2 h pre-incubation of Sal A (25, 50, and 100 μM). LDH release in the culture was determined. **(E–G)** HepG2 cells were treated with 0.5 mM PA for 12 h with or without 2 h pre-incubation of 100 μM Sal A. Cell death was detected by nuclear morphology observation with Hoechst staining using fluorescence microscopy at a magnification of ×200, propidium iodide (PI) staining using flow cytometry, and MTT test, respectively. All values are denoted as means ± SD from three or more independent batches of cells. The values with different superscripts are significantly different at *p* < 0.05. # Comparisons with normal control group; *Comparisons with individual PA treatment groups.

We next investigated the anti-lipotoxicity potential of Sal A using HepG2 cells. The cytotoxicity of Sal A was first evaluated by MTT test. As shown in Figure 3A, it was only above 750 μM that Sal A showed obvious cytotoxicity. The *in vitro* lipotoxicity model was established by palmitic acid exposure of HepG2 cells. Our results indicated that palmitic acid exposure triggered cell death in HepG2 cells in both dose-dependent and time-course manner (Figures 3B,C). Importantly, Sal A pretreatment prevented palmitic acid-induced cell death, which was confirmed by several measurements, including LDH release, nuclear staining, propidium iodide staining, and MTT test (Figures 3D–G).

The protective role of Sal A is independent of MAPKs and PKA pathways.

**FIGURE 4:**
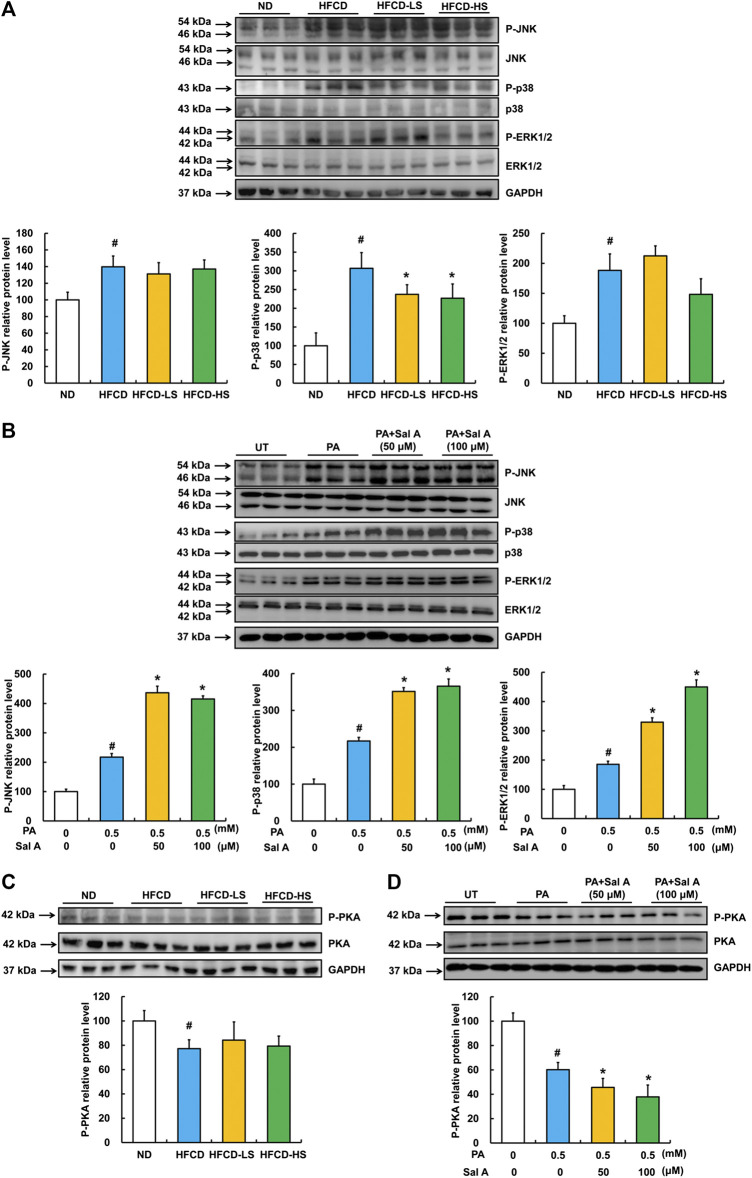
Effects of Sal A administration on p-JNK, p38, p-ERK1/2, and p-PKA in HFCD-fed mice and palmitate-treated hepatocytes. Total cellular lysates were extracted from mice liver tissues. HepG2 cells were exposed to 0.5 mM palmitic acid (PA) for 12 h with or without 2 h pre-incubation of Sal A (50 and 100 μM). **(A,B)** Immunoblotting assay was performed for p-JNK, p-p38, and p-ERK1/2. **(C,D)** Immunoblotting assay was performed for p-PKA. All values are denoted as means ± SD 12 animal liver samples per group (*n* = 12) or at least three independent batches of cells. The values with different superscripts are significantly different at *p* < 0.05. # Comparisons with normal control group; * Comparisons with singly PA treatment group.

Previous studies have reported that the activation of MAPKs, including JNK, p38, and ERK1/2, were involved in palmitic acid-induced lipotoxicity (Liu et al., 2018; Liu et al., 2015; Yuan et al., 2010). Accordingly, we assessed effects of Sal A on JNK, p38, and ERK1/2. Our data revealed that phosphorylation of all above three MAPKs was increased by HFCD-feeding, while Sal A only significantly reversed HFCD-induced p-p38 upregulation (Figure 4A). Moreover, exposure to palmitic acid markedly increased phosphorylation of JNK, p38, and ERK1/2 abundance, whereas Sal A treatment did not reverse the effects of palmitic acid on these kinases (Figure 4B). In addition, we previously documented that the prevention of suppression of PKA by palmitate was beneficial in protecting against lipotoxicity (Li et al., 2015). Consistently, p-PKA was significantly inhibited in both HFCD-fed mice and palmitic acid-treated hepatocytes. However, Sal A did not improve p-PKA suppression (Figures 4C, D), excluding its contribution to Sal A’s protective effect on lipotoxicity.

AMPK activation contributes to Sal A’s anti-lipotoxic effect in HepG2 cells.

**FIGURE 5:**
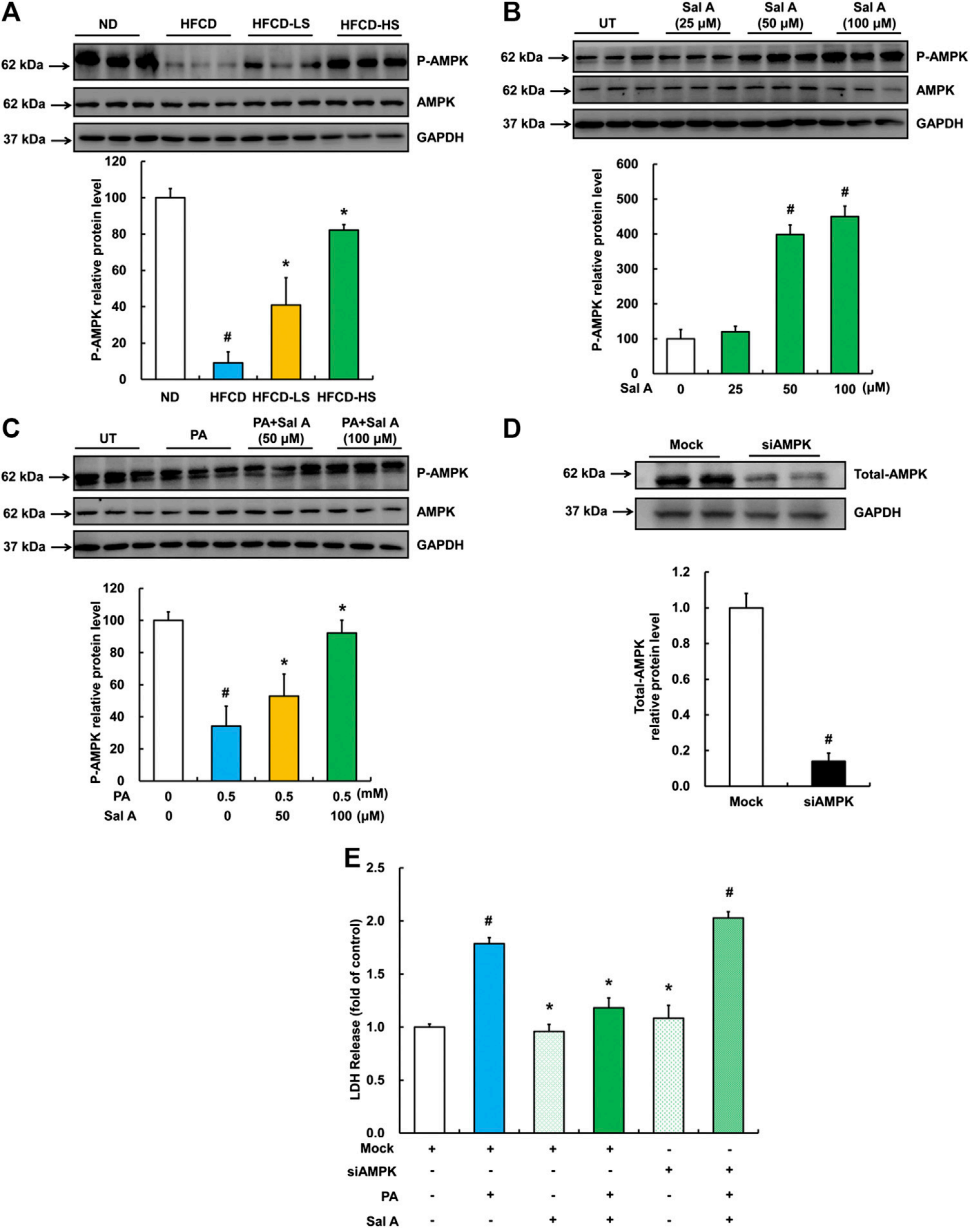
Sal A-activated AMPK contributed to the protection against lipotoxicity-induced liver injury. **(A)** Total cellular lysates were extracted from mice liver tissues. Immunoblotting was performed for p-AMPK. **(B)** Cells were treated with different doses of Sal A (25, 50, and 100 μM) for 12 h p-AMPK was detected. **(C)** HepG2 cells were exposed to 0.5 mM palmitic acid (PA) for 12 h with or without 2 h pre-incubation of Sal A (50 and 100 μM). p-AMPK was detected. **(D)** HepG2 cells were transfected with siRNAs for AMPK. Silencing efficiency was detected by Immunoblotting assay for AMPK expression. **(E)** After siRNA silencing of AMPK, cells were exposed to 0.5 mM PA for 12 h with or without 2 h Sal A (100 μM) pretreatment. LDH release was detected. All values are denoted as means ± SD from 12 animal liver samples per group (*n* = 12) or at least three independent batches of cells. The values with different superscripts are significantly different at *p* < 0.05. # Comparisons with normal diet (ND) or normal control group; * Comparisons with HFCD group or singly PA treatment group.

AMPK activation protects against lipotoxicity (Minokoshi et al., 2002; Wang et al., 2007; Wu et al., 2013a). We previously reported that palmitic acid exposure suppressed AMPK activity in HepG2 cells (Li et al., 2017). To determine if AMPK is a target of Sal A, we subsequently examined the regulation of Sal A on hepatic p-AMPK abundance in both HFCD-fed mice and palmitic acid-exposed HepG2 cells. Our results indicated that Sal A administration rescued HFCD-induced reduction of hepatic p-AMPK abundance (Figure 5A). *In vitro*, Sal A activated AMPK in a dose-dependent manner (Figure 5B). Importantly, the Sal A pretreatment alleviated palmitic acid-induced decrease of p-AMPK protein abundance (Figure 5C). To confirm that the prevention of palmitic acid-induced AMPK inhibition was involved in the protective role of Sal A against lipotoxicity, AMPK gene silencing experiment with siRNA transfection was conducted (the siRNA knockdown efficiency shown in Figure 5D). Our result clearly showed that silencing AMPK abolished the protective role of Sal A (Figure 5E), indicating that retaining AMPK activity contributes to Sal A’s anti-lipotoxicity property.

SIRT1 upregulation contributes to Sal A’s hepatoprotective action against lipotoxicity.

**FIGURE 6:**
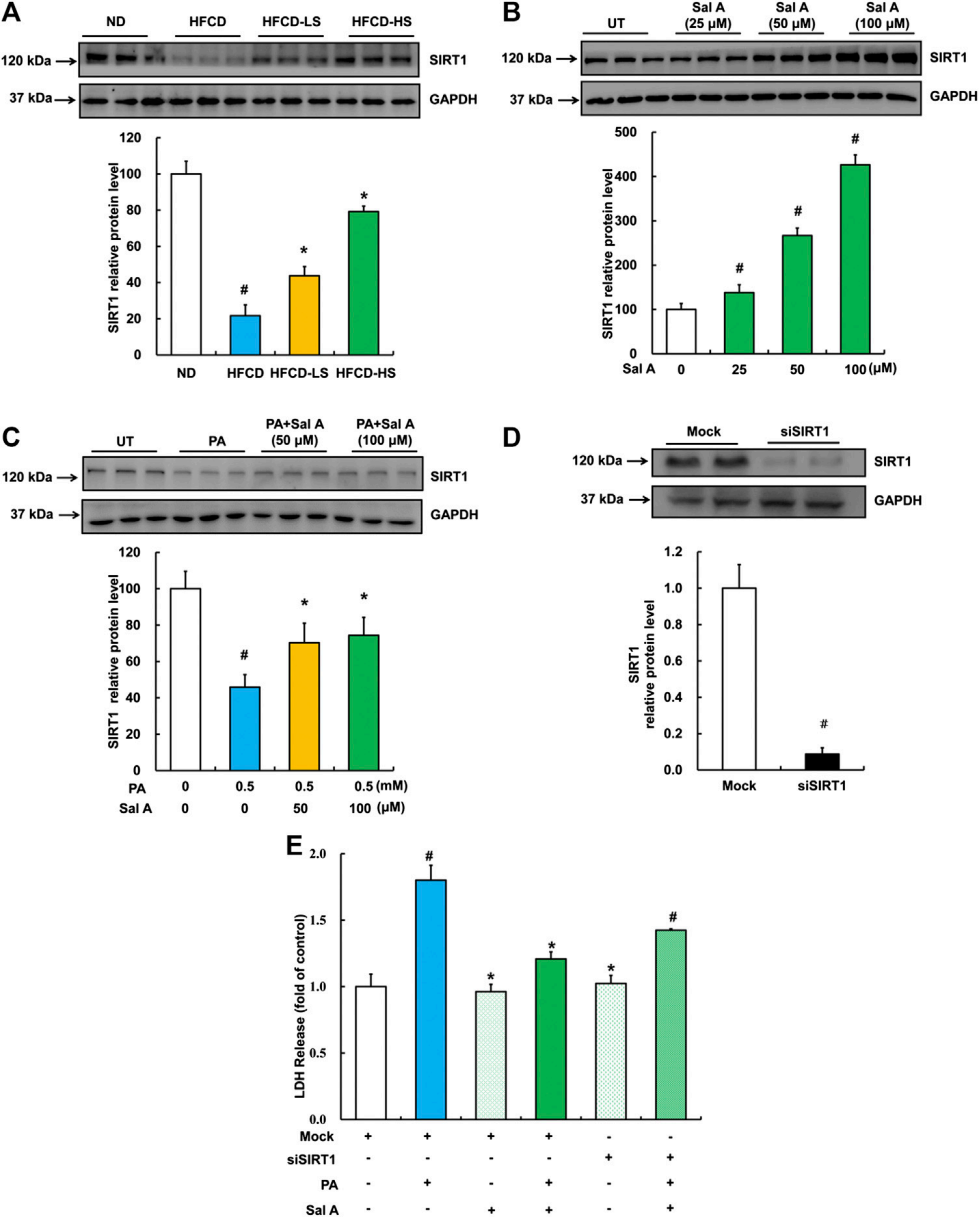
Upregulation of SIRT1 by Sal A protected against lipotoxicity-induced liver injury. **(A)** Total cellular lysates were extracted from mice liver tissues. Immunoblotting was performed for SIRT1. **(B)** Cells were treated with different doses of Sal A (25, 50, and 100 μM) for 12 h. SIRT1 expression was detected. **(C)** HepG2 cells were exposed 0.5 mM palmitic acid (PA) for 12 h with or without 2 h pre-incubation of Sal A (50 and 100 μM). SIRT1 was detected. **(D)** HepG2 cells were transfected with siRNAs for SIRT1. Silencing efficiency was detected by Immunoblotting assay for SIRT1 expression. **(E)** After silencing SIRT1 by siRNA, cells were exposed to 0.5 mM PA for 12 h with or without 2 h Sal A (100 μM) pretreatment. LDH release was detected. All values are denoted as means ± SD from 12 animal liver samples per group (*n* = 12) or at least three independent batches of cells. The values with different superscripts are significantly different at *p* < 0.05. # Comparisons with normal diet (ND) or normal control group; * Comparisons with HFCD group or singly PA treatment group.

We previous reported that activating SIRT1 improved palmitic acid-induced cell death in hepatocytes (Shen et al., 2017). Therefore, we next examined hepatic SIRT1 expression using liver samples from our animal studies. As shown in Figure 6A, HFCD-feeding led to a robust decline of liver SIRT1 expression, which was significantly blunted by Sal A administration. In HepG2 cells, Sal A enhanced SIRT1 expression in a dose-dependent manner (Figure 6B). Importantly, HepG2 cells pretreated with Sal A demonstrated a marked improvement of palmitic acid-induced SIRT1 downregulation (Figure 6C). Similarly, SIRT1 gene silencing through siRNA transfection blocked the protective role of Sal A (Figure 6D,E), which indicated that SIRT1 was mechanistically involved in the protection of Sal A against hepatic lipotoxicity.

SIRT1 is the downstream target of Sal A-triggered AMPK activation.

**FIGURE 7:**
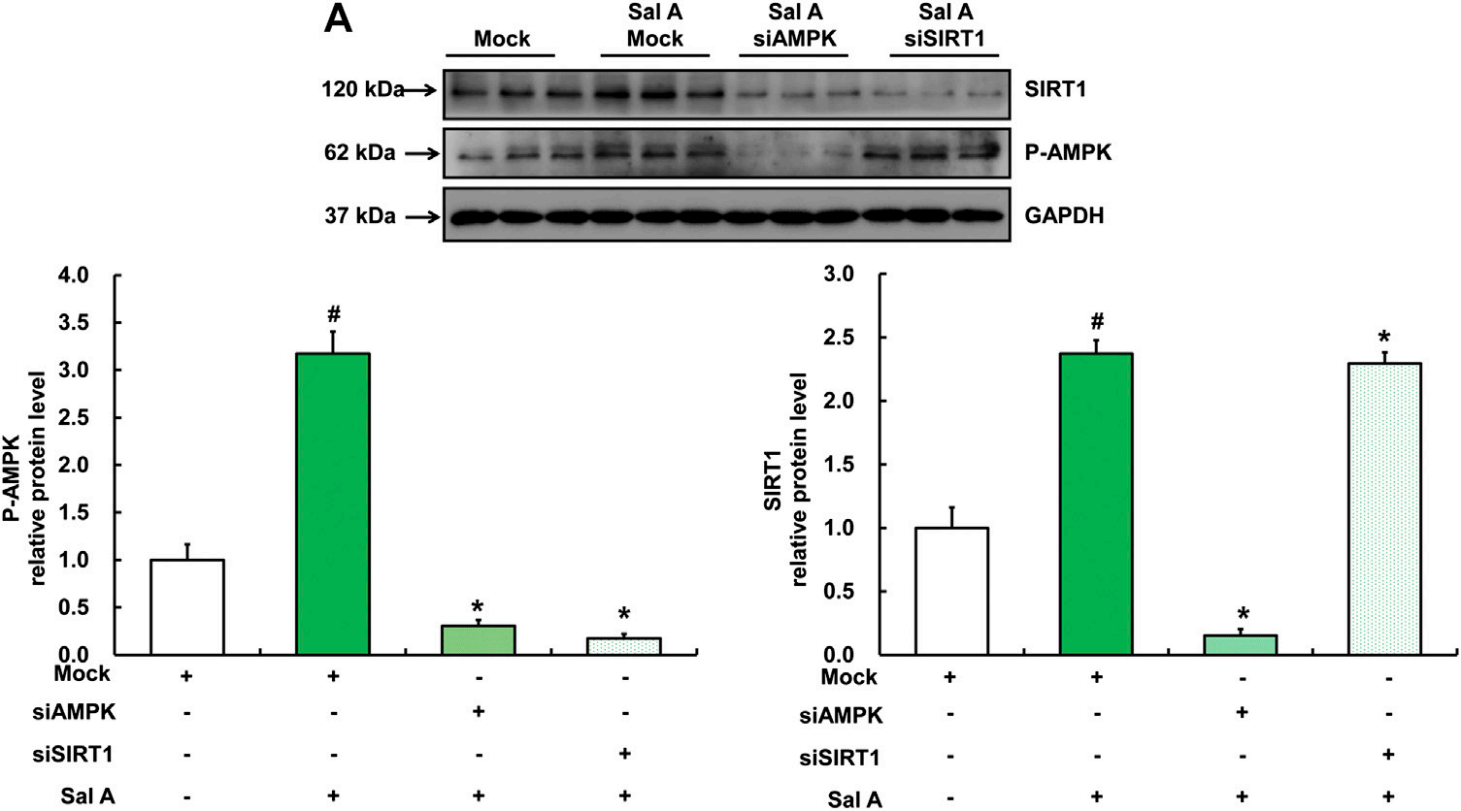
SIRT1 was downstream of Sal A-activated AMPK pathway. HepG2 cells were transfected with siRNA for SIRT1 or AMPK, respectively. Then cells were treated with Sal A (100 μM) for 12 h. Total cellular lysates were collected for the immunoblotting assay for SIRT1 and p-AMPK. All values are denoted as means ± SD from three or more independent batches of cells. The values with different superscripts are significantly different at *p* < 0.05. # Comparisons with control group; * Comparisons with singly Sal A treatment group.

We next analyzed the mutual regulatory relationship between AMPK and SIRT1 by gene silencing. Our results showed that silencing AMPK significantly inhibited Sal A-activated SIRT1 up-regulation, whereas silencing SIRT1 did not affect the activation of p-AMPK by Sal A (Figure 7), suggesting that SIRT1 is a downstream target in Sal A-induced AMPK activation.

## Discussion

The present study demonstrates for the first time that Sal A, a natural polyphenolic compound extracted from *Radix Salvia miltiorrhiza* (Danshen), confers protection against hepatic lipotoxicity in both HFCD-fed mice and cultured hepatocytes. The results in this study uncover that Sal A exerts its beneficial role via activating the AMPK-SIRT1 signal pathway.

Sal A has been recognized as an important bioactive ingredient from *Radix Salvia miltiorrhiza*, which is a traditional herb in China, and is widely used as functional food for the treatment and prevention of cardiovascular diseases (Song et al., 2015). More recently, evidence has been emerging that Sal A possesses hepatic protective property. Ding et al. has reported that Sal A gavage (8 and 16 mg/kg/d) ameliorated HFD-induced NAFLD in rats (Ding et al., 2016). In this study, we tested the effect of Sal A on HFCD-induced NAFLD in C57BL/6 mice. The selected doses of Sal A for animal study were referred from a previous report (Sui et al., 2007; Shen et al., 2009). Sal A was injected every two days to reduce the incidence of abdominal infection. Our results clearly demonstrated that Sal A injection alleviated HFCD-induced body weight gain, hepatic steatosis, and liver injury. Although we did not investigate here the effects of Sal A on glycometabolism, its beneficial role in alleviating insulin resistance has been reported (Qiang et al., 2015). We did not observe any effects of Sal A on food intake, skin color, hair count, and daily behavior. Notably, Sal A clearly improved HFCD-reduced mobility in mice. Under ideal conditions, the estimated peak concentration of Sal A in mice blood were 0.69 and 1.38 mM in HFCD-LS (20 mg/kg BW) group and HFCD-HS (40 mg/kg BW) group, respectively. Although there is no evidence of clinical application of salvianolic acid A alone, Salvianolic acids for injection (SAI) is a clinical used medicinal preparation composed of multiple salvianolic acids from the aqueous extracts of Danshen. The concentration of Sal A in SAI is 72 μg/ml (Zhang et al., 2013). We estimated that the concentration of Sal A in the blood of a human body is about 0.65 μM with a recommended dosage of 20 ml/d via intravenous injection. The concentration of Sal A in Danshen is 0.14 mg/g (Luo et al., 2015). Considering the oral dose of Danshen in clinical prescription is 10–30 g, the estimated corresponding concentration of Sal A in the blood of human body is about 0.63–1.89 μM. We can clearly find that the blood concentration of Sal A in humans mentioned above is far lower than that in our experiment.

Lipotoxicity caused by SFAs in hepatocytes plays vital roles in both initiation and progression of NAFLD (Leamy et al., 2013; Perla et al., 2017). Ameliorating liver lipotoxicity had exhibited efficacy in improving HFD-induced NAFLD (Li et al., 2017). Therefore, searching effective drugs or compounds from natural herbs that protect against lipotoxicity is commonly considered as an ideal therapeutic strategy for the treatment of NAFLD. Although Sal A exhibited cytoprotective effects against different stimuli in various cell types, to the best of our knowledge, few studies have addressed the protective role of Sal A against SFAs-induced hepatocyte cytotoxicity. Sal A also attenuated angiotensin Ⅱ-induced apoptosis in murine peritoneal macrophages (Li et al., 2016) and protected cardiomyocytes from toxicity induced by arsenic trioxide, via inhibition of the MAPK pathway (Zhang et al., 2017). Furthermore, Sal A improved ischemia-reperfusion-stimulated injury in brain, renal, and intestines (Hou et al., 2016; Song et al., 2018; Zu et al., 2018). In the liver, Sal A significantly reduced chronic alcohol feeding- or carbon tetrachloride-induced liver injury in rats (Wu et al., 2007; Shi et al., 2018a). In the present study, we reported for the first time that Sal A alleviated palmitic acid-induced hepatic cytotoxicity and improved liver pathologies in HFCD-fed mice. A wide-range of doses of Sal A from 1 nM to 100 μM have been used in previous cell culture studies (Qiang et al., 2015; Li et al., 2019). In this study, ascending doses of Sal A (25, 50, and 100 μM) were chosen based on our observations that no further protections were observed when Sal A concentrations higher than 100 μM were used (Supplementary Figure S1).

The potential mechanisms underpinning the Sal A anti-lipotoxic action in hepatocytes remain elusive. Lipotoxicity-induced MAPKs activation has been documented to involve its cytotoxicity effect (Srivastava et al., 2007). Recent research revealed the inhibitory role of Sal A on MAPKs in various cells (Dai et al., 2015; Zhang et al., 2017; Feng et al., 2020). Thus, here, we determined the effects of Sal A on MAPKs in hepatocytes. All three MAPKs, including p-JNK, p-p38, and p-ERK1/2, were stimulated by HFCD administration, whereas Sal A intervention only reversed p-p38 induction in mice. This implies that p38 MAPK is a potential target of Sal A against HFCD-induced liver injury. Unexpectedly, Sal A treatment did not protect but rather aggravated MAPKs induction by palmitate in HepG2 cells. Although the exact reason was not clear, previous findings demonstrated that Sal A possessed anti-tumor ability and exhibited a dual-directional regulation between normal and tumor cells on signal transduction (e.g., p-Akt) (Pei et al., 2018; Li et al., 2019). Since limited work has reported the regulatory role of Sal A on MAPKs in hepatocytes, further study should be performed in non-oncogenic hepatocytes. We also analyzed the effect of Sal A on HFCD- and palmitate-induced PKA inhibition, which also contributes to hepatic cytotoxicity (Li et al., 2015). Our data showed that Sal A intervention did not reverse p-PKA suppression. However, we could not exclude the participation of PKA signal, since several studies revealed that Sal A stimulated cAMP/PKA pathway, and hence protected against arterial thrombosis and vascular smooth muscle injury, respectively (Sun et al., 2016; Zhao et al., 2017). Therefore, the measurement of hepatic cAMP level under Sal A exposure is still needed in further studies. AMPK, a key sensor of energy metabolism, plays an important role in regulating lipotoxicity. Our previous studies have reported that AMPK activation could significantly eliminate lipotoxicity-induced cell death in hepatocytes (Li et al., 2014; Li et al., 2017). In the present study, our data revealed that Sal A treatment reversed HFCD- and palmitate-inhibited AMPK phosphorylation. More importantly, genetically silencing AMPK blocked the protective role of Sal A, indicating the mechanistic involvement of the AMPK pathway. In agreement with our results, Sal A has also been reported to activate AMPK in the liver of diabetic rats (Yu et al., 2012; Qiang et al., 2015).

The potential mechanisms linking Sal A-regulated AMPK activation are still unclear. Commonly, AMPK is stimulated by two classical signals. One is Ca^2+^-dependent pathway, which is mediated by calcium/calmodulin-dependent protein kinase kinase β (CaMKKβ) (Fogarty et al., 2010); and the other one is AMP-dependent pathway, which is regulated by liver kinase B1 (LKB1) (Gan and Li, 2014). Although we did not directly measure the intracellular AMP/ATP ratio under Sal A treatment, Qiang et al. reported that Sal A increased intracellular ATP level in HepG2 cells (Qiang et al., 2015). Sal A stimulated AMPK activation in the absence of LKB1 in HeLa cells (Qiang et al., 2015). These evidences indirectly excluded the role of AMP/ATP regulated LKB1 in Sal A-activated AMPK. Although limited studies have reported the regulation of Sal A on intracellular calcium concentration in hepatocytes, recent evidence confirmed that CaMKKβ inhibitors could significantly block Sal A-induced AMPK activation in HeLa cells (Qiang et al., 2015), implying that a Ca^2+^-dependent pathway may contribute to Sal A-induced AMPK activation.

SIRT1, a NAD^+^-dependent deacetylase, is predominantly localized in the nucleus. SIRT1 activation exhibited various beneficial biofunctions, including extending lifespan, delaying senescence, and improving metabolic diseases, such as NAFLD, diabetes, and obesity (Mariani et al., 2015; Cao et al., 2017; Kumar et al., 2017). We previously reported that SIRT1 activation was mechanistically involved in the protection against hepatic lipotoxicity (Li et al., 2015). SIRT1 activation prevented alcohol-, cholestasis-, and concanavalin A-induced liver injury (Xu et al., 2013; Kulkarni et al., 2016; Shi et al., 2018a). Therefore, we investigated the involvement of SIRT1 in the anti-lipotoxicity role of Sal A in this study. Our results clearly indicated that Sal A-increased SIRT1 expression contributed to the protective role of Sal A. Recent studies revealed the mutual regulatory relationship between AMPK and SIRT1 (Canto et al., 2009; Ganesan et al., 2017). However, the cross-talk between AMPK and SIRT1 under the exposure of Sal A is still elusive. We further tested the reciprocal relationship between AMPK and SIRT1 under Sal A treatment. We observed that AMPK was involved in Sal A-regulated SIRT1 based on the listed evidence: 1) AMPK silencing significantly blocked Sal A-increased SIRT1 expression; otherwise, SIRT1 silencing did not affect Sal A-upregulated p-AMPK level; 2) while genetically silencing SIRT1 blocked about 40% of the protective role of Sal A, silencing AMPK completely inhibited Sal A-protected lipotoxicity. It means that, besides SIRT1, there probably exist other downstream targets of AMPK. Recent studies have identified thioredoxin-interacting protein (TXNIP), which could be degraded by AMPK activation and involved in Sal A-treated rats, and might be another potential target (Wu et al., 2013b; Ding et al., 2016; Wei et al., 2019).

The current strategies for treating NAFLD can be summarized into four aspects, to improve: 1) hepatic steatosis and the consequent metabolic stress; 2) oxidative stress, inflammation, and cell damage or apoptosis; 3) disturbance of intestinal flora; and 4) fibrinolytic pathway. Here, we concluded the potential targets of Sal A based on the current literature and our study. First, we observed that Sal A treatment significantly rescued oleic acids-induced lipids deposition in cultured hepatocytes (Supplementary Figure S2). In support of our finding, Sal A exhibited a strong ability against hepatic steatosis in both energy-enriched diet-fed rats and palmitic acid-treated hepatocytes (Ding et al., 2016). Second, recent studies have demonstrated that Sal A strongly mitigates oxidative stress (Wu et al., 2007; Jiang et al., 2008; Tsai et al., 2010; Zhang et al., 2014; Zu et al., 2018), a mechanism involved in palmitate-induced cell death (Li et al., 2014). Activation of nuclear factor (erythroid-derived 2)-like 2 (Nrf2) facilitated Sal A-mediated to oxidative stress in different experimental settings (Zhang et al., 2014; Gu et al., 2017; Zhang et al., 2017). Therefore, Sal A-stimulated Nrf2 activation might be involved in the protection against NAFLD via anti-oxidative stress. Additionally, the anti-inflammatory role of Sal A has been reported in renal, brain, and cardiac tissues (Yuan et al., 2017; Zhang et al., 2018; Zhang et al., 2019). A recent study revealed that Sal A supplementation alleviated HFD-induced hepatic inflammation (Ding et al., 2016). In cultured hepatocytes, Sal A prevented TNF-alpha-induced apoptosis (Yan et al., 2015). To our knowledge, limited studies have addressed the beneficial effects of Sal A on intestinal microecological and fibrinolytic pathway in NAFLD. The existing evidence indicates that Sal A exerts its protective role against NAFLD by targeting hepatic steatosis, oxidative stress, inflammation, and lipotoxicity-related liver damage.

The main finding of the present study was the anti-lipotoxic effects of Sal A to reduce hepatocyte apoptosis. We previously confirmed that the activation of autophagy prevented hepatocytes against SFAs-induced cytotoxicity (Li et al., 2017; Li et al., 2014). Accumulating evidence supported that the activation of both AMPK and SIRT1 stimulated autophagy (Yan et al., 2017). Moreover, recent evidence demonstrated that Sal A promoted autophagosome-lysosome fusion (Shi et al., 2018b). We observed that Sal A treatment significantly reversed the reduction of an important autophagy marker, LC3-II, in livers from HFCD a special marker of autophagy (Supplementary Figure S3). We therefore presumed that autophagy activation was probably involved in Sal A protected lipotoxicity via activating AMPK/SIRT1 pathway. A limitation of this study is the lack of a positive control drug. Although there is no FDA approved drug for NAFLD so far, some ongoing clinical trials using statins, metformin, and pioglitazone are potential positive controls to objectively evaluate the effect of Sal A in the future studies (Patel and Siddiqui, 2019).

In summary, we reported for the first time that Sal A protects against hepatic lipotoxicity by activating the AMPK-SIRT1 signaling pathway. Our findings suggest that Sal A is a potential candidate for the treatment of NAFLD and other metabolic disorders with lipotoxicity as an underlying pathological condition.

## Data Availability Statement

The raw data supporting the conclusions of this article will be made available by the authors, without undue reservation, to any qualified researcher.

## Ethics Statement

The animal study was reviewed and approved by Ethics Committee of Zhejiang Chinese Medical University.

## Author Contribution

XD and HC conceptualized the study. SL, QQ, LF, and FJ contributed to the animal experiments. QQ, NY, JL, SZ, and QS contributed to the cell experiments. NY contributed to the data collection and statistical analysis. HC contributed to the interpretation of experimental results. SL and QQ drafted the manuscript. XD and SL finalized the manuscript.

## Funding

This work was supported by the National Natural Science Foundation of China (grant number: 81973041 & 81773981); Zhejiang Natural Science Foundation for Distinguished Young Scholars (grant number: LR20H260001); Research Fund of Zhejiang Chinese Medical University (grant number: 2020ZZ09).

## Conflict of Interest

The authors declare that the research was conducted in the absence of any commercial or financial relationships that could be construed as a potential conflict of interest.
