# Supplementary material for: Activation of the AMPK-SIRT1 pathway contributes to protective effects of Salvianolic acid A against lipotoxicity in hepatocytes and NAFLD in mice
Source: Front Pharmacol. 2020 Nov 30;11:560905. doi: 10.3389/fphar.2020.560905 (PMC7734334; doi:10.3389/fphar.2020.560905)
Supplement: Supplementary file 1 [file datasheet1.docx]

Supplementary Data

Figure S1


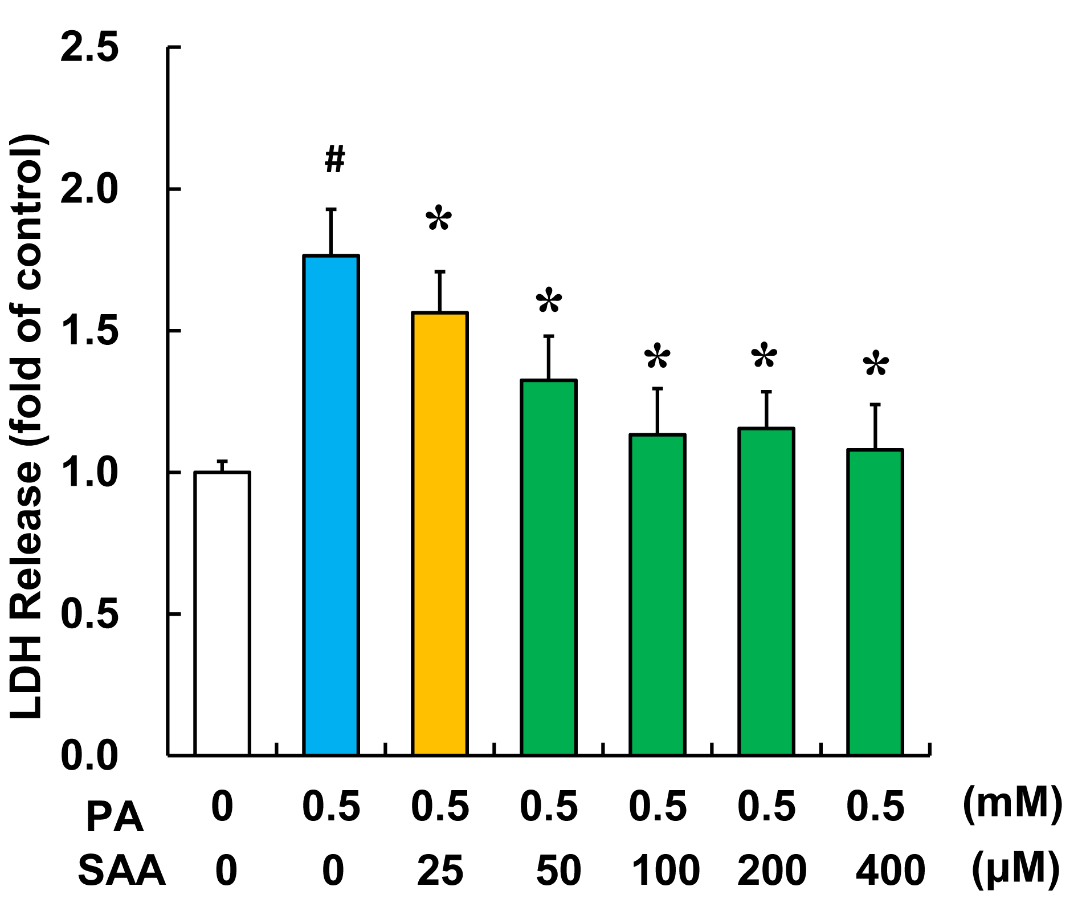


Figure S1 Sal A protected hepatocytes against palmitate acids-induced cell death. HepG2 cells were treated with 0.5 mM palmitic acid (PA) for 12 h with or without 2 h pre-incubation of Sal A (25, 50, 100, 200, and 400 μM). LDH release in the culture was determined. All values are denoted as means ± SD from three or more independent batches of cells. The values with different superscripts are significantly different at *p* ˂ 0.05. # reflects comparing with normal control group; * reflects comparing with singly PA treatment group.

Figure S2


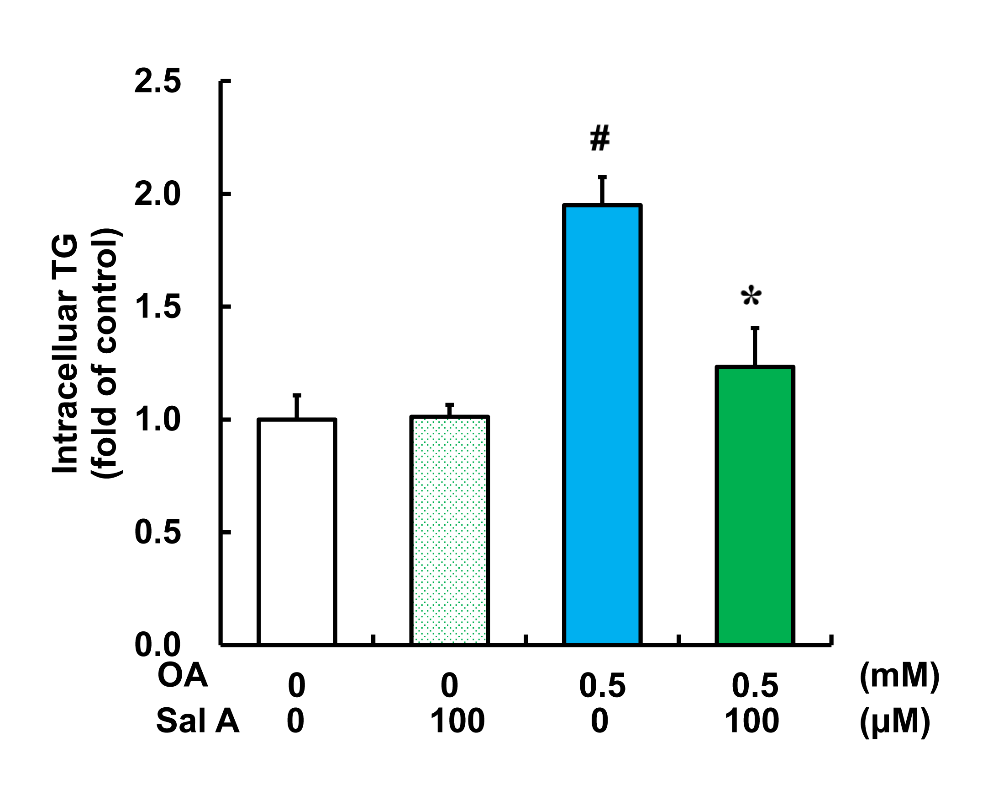


Figure S2 Salvianolic acid A improved oleate-induced lipids accumulation in HepG2 cells. HepG2 cells were treated with oleic acids (OA, 0.5 mM) for 12 h. Salvianolic acid A (Sal A, 100 μM) was added 1 h before OA treatment. Intracellular lipids deposition was determined by the measurement of triglyceride (TG). For intracellular TG detection, cells were collected and lysed. The contents of TG and protein in lysate were determined by TG assay kit (Nanjing Jiancheng Bioengineering Institute, Nanjing, China) and BCA kit (Beyotime, Shanghai, China), respectively, according to the manufacturer’s instructions. The ratio of TG level to protein concentration was calculated to express the relative TG content in cells. All values are denoted as means ± SD from three independent batches of cells. The values with different superscripts are significantly different at *p* ˂ 0.05. # reflects comparing with control group; * reflects comparing with singly OA treatment group.

Figure S3


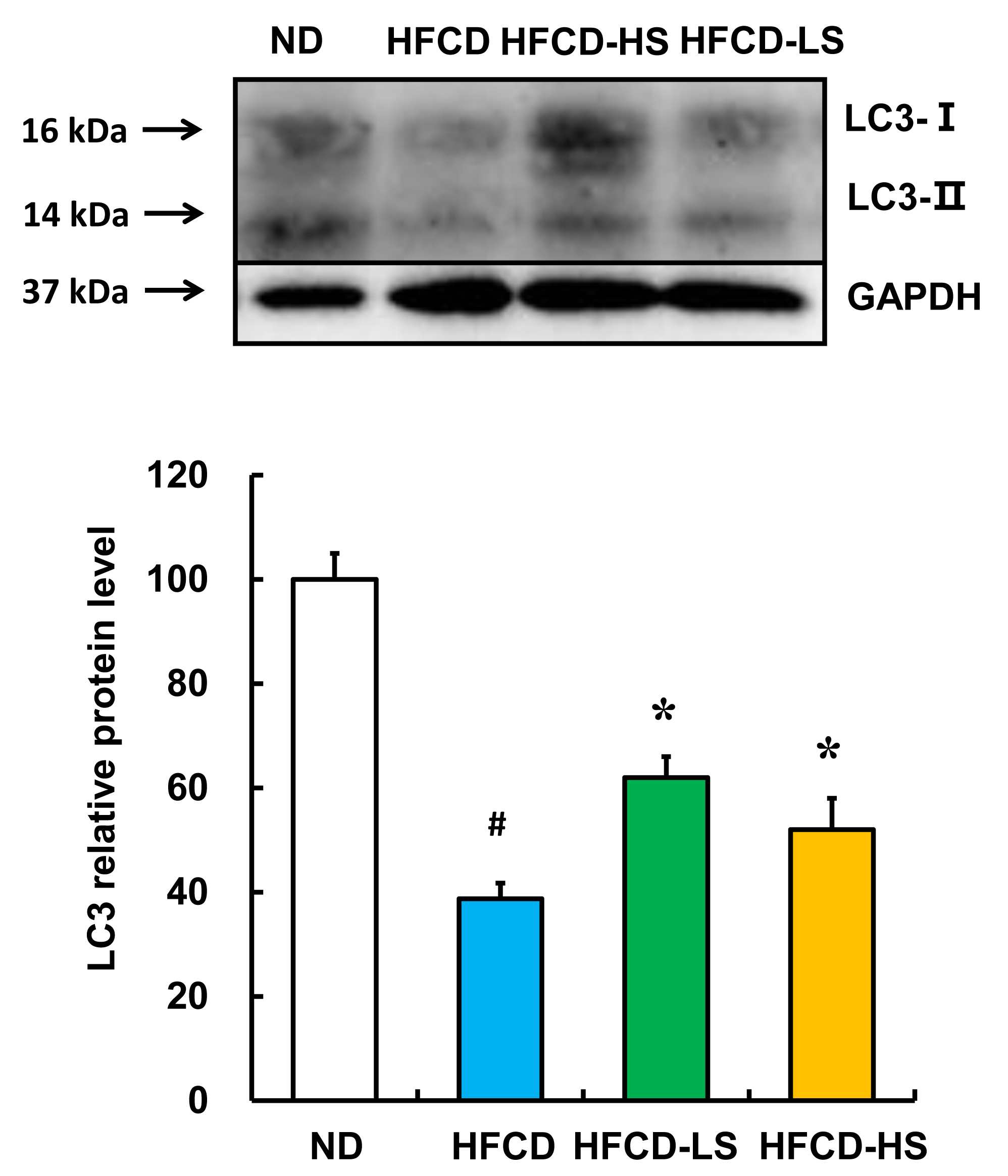


Figure S3 Salvianolic acid A treatment ameliorated HFCD-inhibited LC3-II expression in liver. Total cellular lysates were extracted from mice liver tissues. Immunoblotting assay was performed for LC3-II. Anti-LC3-II antibody was purchased from Cell Signaling Technology (Danvers, MA). All values are denoted as means ± SD from 12 animal liver samples per group (n = 12). The values with different superscripts are significantly different at *P* ˂ 0.05. # reflects comparing with normal diet (ND) group; * reflects comparing with high-fat and high-carbohydrate diet (HFCD) group.
